# Scalable Fabrication of Biomimetic Antibacterial Nanospikes on PMMA Films Using Atmospheric-Pressure Low-Temperature Plasma

**DOI:** 10.3390/biomimetics10090601

**Published:** 2025-09-08

**Authors:** Masashi Yamamoto, Kentaro Tada, Ayumu Takada, Atsushi Sekiguchi

**Affiliations:** 1 Department of Electrical and Computer Engineering, National Institute of Technology, Kagawa College, 335 Chokushi-cho, Takamatsu 761-8058, Japan; 2 Litho Tech Japan Corporation, 2-6-6 Namiki, Kawaguchi 332-0034, Japan; 3 Graduate School of Engineering, Division of Physics and Electronics, Osaka Metropolitan University, 3-3-138 Sugimoto-cho, Sumiyoshi-ku, Osaka 558-8585, Japan

**Keywords:** scalable antibacterial surface fabrication, atmospheric-pressure low-temperature-plasma large-area surface treatment, polymer surface engineering, nanospike-structured surfaces

## Abstract

Antibacterial surfaces inspired by biological micro- and nanostructures, such as those found on the wings of cicadas and dragonflies, have attracted interest due to their ability to inhibit bacterial adhesion and damage microbial membranes without relying on chemical agents. However, conventional fabrication techniques like photolithography or nanoimprinting are limited by substrate shape, size, and high operational costs. In this study, we developed a scalable method using atmospheric-pressure low-temperature plasma (APLTP) to fabricate sharp-edged nanospikes on solvent-cast polymethyl methacrylate (PMMA) films. The nanospikes were formed through plasma-induced modification of pores in the film, followed by annealing to control surface wettability while maintaining structural sharpness. Atomic force microscopy confirmed the formation of micro/nanostructures, and contact angle measurements revealed reversible hydrophilicity. Antibacterial performance was evaluated against *Escherichia coli* using ISO 22196 standards. While the film with only plasma treatment reduced bacterial colonies by 30%, the film annealed after plasma treatment achieved an antibacterial activity value greater than 5, with bacterial counts below the detection limit (<10 CFU). These findings demonstrate that APLTP offers a practical route for large-area fabrication of biomimetic antibacterial coatings on flexible polymer substrates, holding promise for future applications in healthcare, packaging, and public hygiene.

## 1. Introduction

Biomimetics, the practice of drawing inspiration from nature, has received increasing attention in recent years, particularly in the development of antibacterial surfaces. Numerous studies have demonstrated that micro- and nanostructured surface topographies inspired by biological organisms can effectively inhibit bacterial adhesion and exert bactericidal effects by physically disrupting bacterial membranes [1,2,3,4,5,6,7,8,9,10]. The nanopillar structures found on cicada wings are known to promote a Cassie–Baxter wetting state [11], which reduces bacterial colonization, while their nanoscale protrusions can mechanically rupture bacterial membranes [1,4,5,6,12,13,14,15]. The structures exhibit antibacterial activity not only due to their chemical property but also due to their physical topography [1,3,10,16,17]. Artificial surfaces such as black silicon, fabricated using reactive ion etching, have been shown to replicate similar bactericidal effects [2,18,19,20,21,22]. In particular, nanostructured surfaces that combine superhydrophobicity with mechanical bactericidal activity are of great interest for biomedical applications, where preventing bacterial adhesion and biofilm formation is critical.

Various fabrication techniques have been employed to replicate such natural architectures [23,24,25,26,27,28,29,30,31,32], including photolithography [3,33,34], nanoimprinting [26,35,36], plasma-based methods [37,38,39], and laser processing [18,40,41]. Photolithography enables sub-100 nm patterning with high precision [42,43,44,45,46,47]. However, both techniques impose limitations on substrate size and shape and require dedicated equipment, which restricts their practicality for large-scale or irregularly shaped surfaces. Plasma-based processes [48,49,50,51,52] often require vacuum (low-pressure) environments, adding further complexity and cost. Nanoimprinting and laser processes can be used in air environments. However, nanoimprinting has several issues, such as mold durability and root clogging. Laser processes require not only laser equipment but also high-performance optical elements. The number of shots is limited because of the lifetime of the light source and optical elements. Furthermore, the throughput is low due to the scanning process using a pulsed laser. These factors present challenges for implementing such techniques in everyday or industrially scalable settings.

In this study, we focus on atmospheric-pressure low-temperature plasma (APLTP) as a practical and scalable method for fabricating antibacterial microstructures on polymer surfaces. APLTP enables plasma processing under ambient conditions, reduces equipment complexity, and allows for the treatment of large or irregularly shaped substrates [53,54,55,56,57,58]. Our previous work demonstrated that APLTP treatment of solvent-cast polymethyl methacrylate (PMMA) films can produce hydrophilic surfaces through the formation of micro- and nanostructures originating from pores located on and within the film [53]. We also showed that APLTP treatment applied to the surface of polyethylene (PE) sheets and the inner walls of PE tubes resulted in ultra-hydrophilic surfaces with excellent antifouling performance against oil-based contaminants, resembling the functionality observed in snail shells [55,56,57]. While such hydrophilic surfaces are effective in fouling prevention, antibacterial applications often demand the coexistence of both sharp-edged nanostructures and water repellency to prevent bacterial adhesion [2,18,19,20,21,22,58]. To address this requirement, we introduce an annealing process following APLTP treatment. By thermally shrinking the surface and closing microscopic pores, we aim to restore hydrophobicity while preserving the bactericidal microstructure. This dual approach seeks to maintain sharp-edged topography and enhance the surface’s antibacterial performance through controlled wettability.

## 2. Materials and Methods

### 2.1. PMMA Film Preparation

PMMA (Sigma-Aldrich Corp., LLC, St. Louis, MO, USA, *M*_W_ = 15,000) films were prepared by solvent casting in this study. The PMMA was dissolved in ethyl lactate (Wako first Grade; Fujifilm Wako Pure Chemical Corp, Osaka, Japan). Its solution concentration was 30 wt%. The solution was spin-coated onto a glass substrate (1 mm thickness, 76 × 52 mm^2^, S9111; Matsunami Glass Ind. Ltd., Kishiwada, Osaka, Japan) using a spin-coater (K-359 S-1; Kyowa Riken Co., Ltd., Suginami, Tokyo, Japan). The rotation speed of the spin coater was 1800 rpm and the rotation time was 24 s. Blade coating can be used for large-area applications. Pre-baking of the substrate was carried out in an oven (CLO-2AH; Koyo Thermo Systems Co., Ltd., Tenri, Nara, Japan) at 100 °C for 60 s. The initial film thickness was measured using a surface profiler (Surfcom 480A; Tokyo Seimitsu Co., Ltd., Hachioji, Tokyo, Japan). The initial PMMA film thickness was about 5 µm. Forming nanostructures on coated films also has the advantage of not being dependent on the material of the underlying substrate. It can impart antibacterial properties to the surface of substrates that are difficult to micro-machine, such as metals.

### 2.2. Plasma Treatment

**Figure 1:**
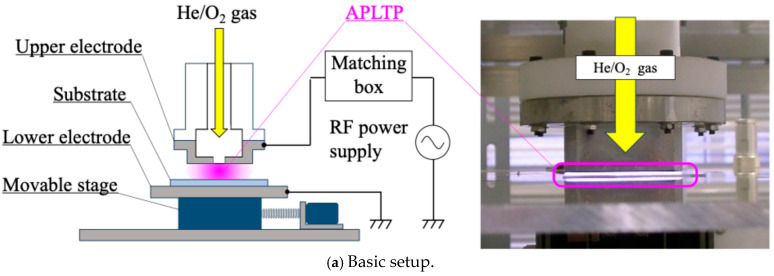

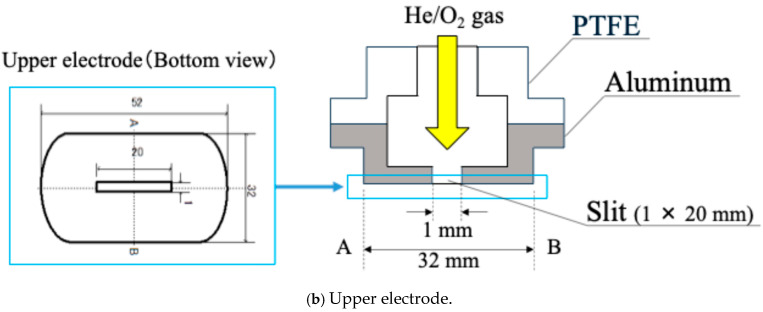
Diagram of APLTP treatment equipment.

The PMMA-coated substrate was irradiated with atmospheric-pressure low-temperature plasma. Figure 1 presents the plasma treatment equipment diagram. The He gas (≥99.99%; Takamatsu Teisan Co., Takamatsu, Kagawa, Japan) flow rate was fixed at 2.0 slm using a mass flow controller (SEC-400MK3; STEC Inc., Toda, Saitama, Japan). The O_2_ gas (≥99.5%; Iwatani Sangyo Corp., Minato, Tokyo, Japan) flow rate was fixed at 60 sccm using another mass flow controller (SEC-E4; HORIBA STEC Co., Ltd., Kyoto, Japan). The He/O_2_ mixture was introduced into an upper electrode. The bottom area of the upper electrode was 32 × 52 mm^2^. The upper electrode had a slit. The slit size was 1 × 20 mm. The mixture flowed out from the slit. The distance between the upper electrode and substrate was 0.7 mm, and the distance between the upper and lower electrode was 1.7 mm. The RF power supply (RP-200-27M; Pearl Kogyo Co., Ltd., Osaka, Japan) and auto matching box (ZDK-916S-2 and M-05A2VD-27M; Pearl Kogyo Co., Ltd., Osaka, Japan) were used to generate an atmospheric-pressure low-temperature plasma. The RF power was 120 W. Its frequency was 27.12 MHz. The stage, on which the lower electrode was placed, could be moved by an electrical motor. The plasma irradiation time per irradiation was 5 s. The cooling time after irradiation was 15 s. This represented one set, which was repeated several times. A small thermocouple (ST-56 K-CA 0200 N-N; RKC Instrument Inc., Otaku, Tokyo, Japan) was used for surface temperature measurement during plasma irradiation. The substrate after plasma treatment was placed in the oven at 120 °C for 5 days [59,60]. This heat treatment process is called annealing herein.

### 2.3. Evaluation of PMMA Film

The relationship between the irradiation cycles of APLTP and the film thickness of PMMA was examined. The plasma resistance of PMMA was evaluated by the etching rate, which was calculated from the decrease in film thickness relative to the irradiation time. The amount of residual solvent in the film was examined using TG-DTA (Rigaku, TG 8121 Thermo plus EVO2, Tokyo, Japan), which combines thermogravimetry (TG) and differential thermal analysis (DTA). The PMMA film prepared on a glass substrate was scraped off with a scraper. The amount recovered was approximately 5 mg. This was placed in an Al pan and heated from room temperature to 500 °C. The heating rate was 20 °C/min. Ar gas (≥99.99%; Takamatsu Teisan Co., Takamatsu, Kagawa, Japan) was flowed at 150 mL/min. The amount of residual solvent was evaluated from the weight loss at 140–200 °C, which is near the boiling point of ethyl lactate.

The surface profile after irradiation was examined using atomic force microscopy (AFM, Veeco Dimension Icon; Veeco Instruments Inc., Plainview, NY, USA). The tip height of the cantilever (Veeco NCHV-W; Veeco Instruments Inc., Plainview, NY, USA) was 10–15 µm, and the tip curvature radius was less than 15 nm. The tip shape was a square pyramid with a forward angle of 25° and a backward angle of 10°. The relation of the surface area to the surface roughness to the plasma irradiation condition was evaluated. The evaluation area of AFM was 5 × 5 μm^2^ with a resolution of 512 × 512 points. The surface roughness was analyzed based on AFM results obtained using WSxM [61]. Before the analysis, each AFM image was digitally treated with parabolic flattening, to suppress three-dimensionality and bow effects.

The wettability of the sample surface was evaluated by contact angle measurements (Simage AUTO 100; Excimer Inc., Yokohama, Japan). The water droplet volume was 2 μL. Pure water (specific resistance value > 18 MΩ·cm) was used for the droplets. The contact angle was measured 1 s after contact of the droplet on the surface. The contact angle was calculated by the tangent method.

The antibacterial test was performed to verify the potential of atmospheric-pressure low-temperature plasma. The antimicrobial performance of the surface was evaluated according to antimicrobial test ISO 22196 using *Escherichia coli* (Escherichia NBRC 3972). *E. coli* was cultured in LB broth with shaking at 30 °C for 24 h. The culture was centrifuged to collect them and suspended in sterile water to a turbidity (OD_600_) of approximately 1.0. This suspension was diluted 100-fold with sterile water to prepare the microbial suspension. An amount of 1.0 mL of this microbial suspension was mixed with 0.2 mL of 10-fold-diluted nutrient bouillon medium and 8.8 mL of sterile water to prepare the test bacterial solution. An amount of 190 µL of the test bacterial solution was dropped onto the surface of the test sample and covered with a 25 × 30 mm^2^ polyethylene film. This area was determined to fit within the upper electrode area. The test bacterial solution and the sample were placed in close contact in a sterilized Petri dish. The polyethylene sheet was used as a control. The Petri dishes containing the samples were left to stand at 35 °C for 24 h and then placed in a sterilized polyethylene bag. An amount of 10 mL of SCDLP medium was added to the bag to wash out the bacteria. The washout solution was serially diluted 10-fold with sterile water, and 0.1 mL of each undiluted solution and diluted solution was applied to a standard agar medium. The medium was left to stand at 35 °C for 48 h to culture the bacteria. After culturing, the number of grown colonies was counted to calculate the viable bacterial count. Since the detection limit in this test was 1 CFU/mL, the lower limit of detection for the number of bacteria per 10 mL of washout solution was 10 CFU. The initial number of colonies was 3.7 × 10^5^ CFU. Three PMMA substrates were prepared under each condition for the antimicrobial test. Only one APLTP treatment condition was applied in this experiment. The test was carried out by an external agency, Osaka Research Institute of Industrial Science and Technology. The condition of *E. coli* on the sample surfaces could not be examined, because of testing constraints.

## 3. Results

### 3.1. Surface Morphology

**Figure 2:**
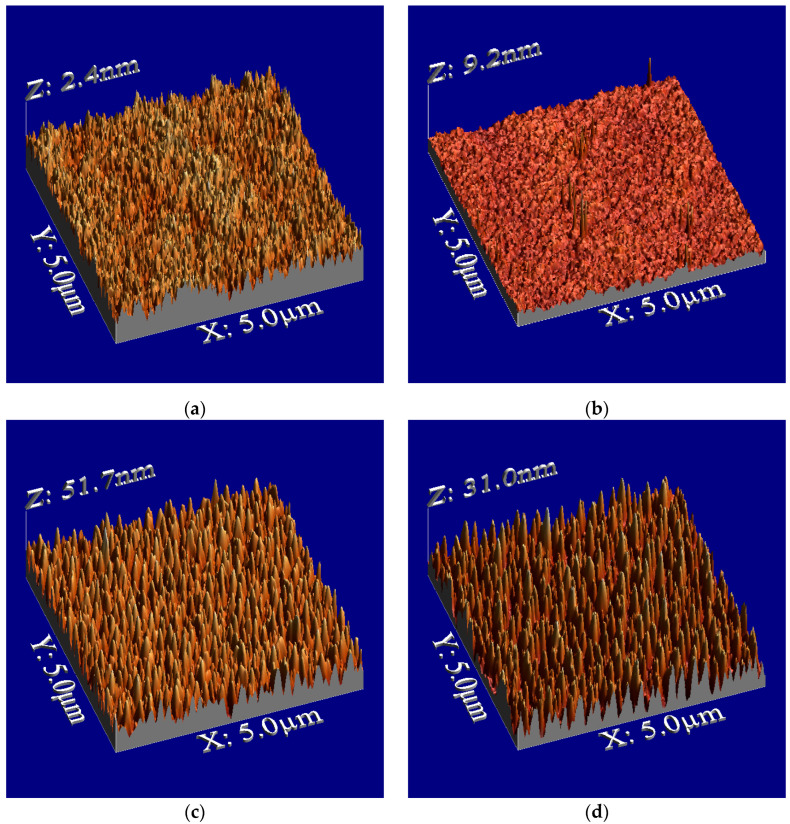
Surface morphologies of PMMA films. (**a**) Untreated PMMA film, (**b**) annealed PMMA film, (**c**) plasma-treated (6 cycles) PMMA film, and (**d**) plasma-treated (6 cycles) and annealed PMMA film.

**Figure 3:**
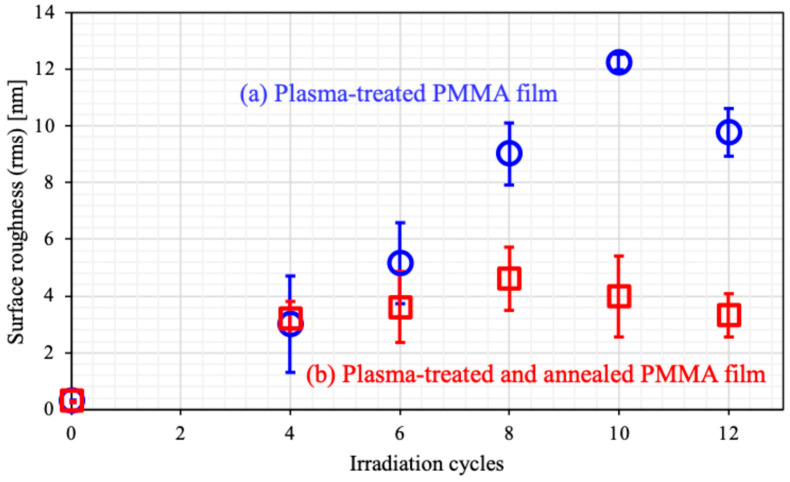
Surface roughness dependence on plasma irradiation times for PMMA films. (**a**) Plasma-treated PMMA film (**○**, blue circle) and (**b**) plasma-treated and annealed PMMA film (**□**, red square).

Figure 2 illustrates the surface morphologies, showing sharp-edged nanospikes on the film surface after plasma treatment. Figure 3 presents the dependence of surface roughness on plasma irradiation cycles for PMMA films. The surface roughness increased up to 10 cycles before decreasing. Irradiation beyond 10 cycles may lead to the destruction of the microstructures. Annealing after plasma treatment resulted in a decrease in the height of the structures, and the surface roughness reduced to about half of the roughness of the plasma-treated surface after the number of irradiation cycles exceeded six. The observed decrease in both the height of the structures and the surface roughness may be attributed to this shrinkage. We discuss thermal shrinking later in this paper.

**Figure 4:**
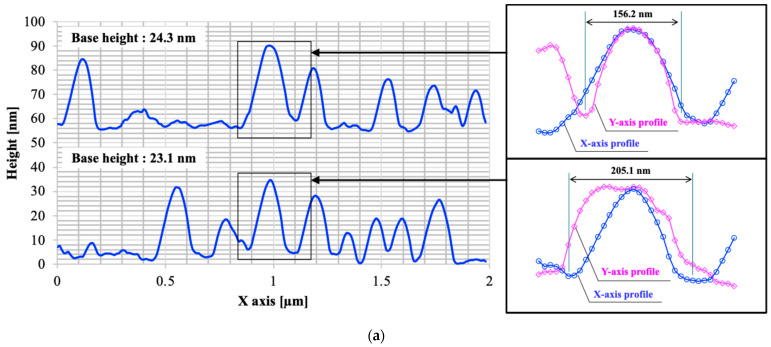

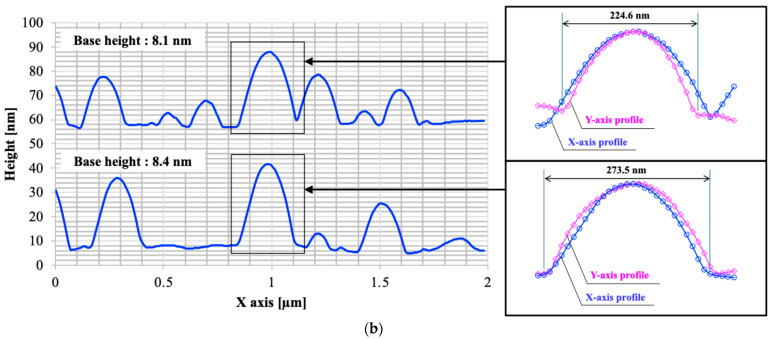
Line profiles of PMMA film surface. (**a**) Plasma-treated (10 cycles) PMMA film and (**b**) plasma-treated (10 cycles) and annealed PMMA film.

The increase in RMS roughness may be influenced by the average height and pitch of the microstructure. Figure 4 illustrates the line profiles of the PMMA film surface after 10 irradiation cycles. As shown in Figure 4, the nanospike height was several tens of nanometers, and the pitch was about 150–200 nm. The substrate surface temperature rose to approximately 150 °C during plasma irradiation with 5 s. This heat must soften the nanospike structure with the increase in irradiation cycles. Neighboring nanospike structures may coalesce, resulting in an increase in pitch. Annealing treatment further flattened the surface. The nanospike height was several tens of nanometers, but the pitch was about 200–300 nm. The average height depends on the base height. A decrease in base height was also observed, which may be ascribed to pore blocking.

The upper limit of RMS roughness may be ascribed to the pores and the process. According to our previous research [53,54], we hypothesize that the microstructure is formed from pores. The pores are thought to be formed by solvent evaporation and are distributed toward the surface. The lower layers become bulk PMMA, making them unsuitable for the formation of microstructures. As the number of irradiations increases, not only does film thickness decrease but the heat effect also increases. Heat causes not only the coalescence of the nanospike structures but also pore sealing, reducing the roughness. They could affect the upper limit of roughness.

### 3.2. Wettability

**Figure 5:**
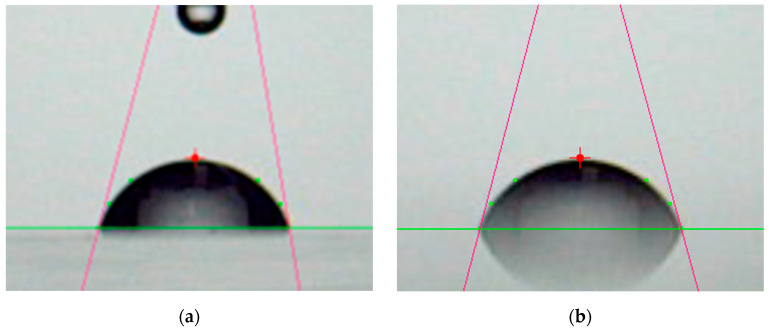

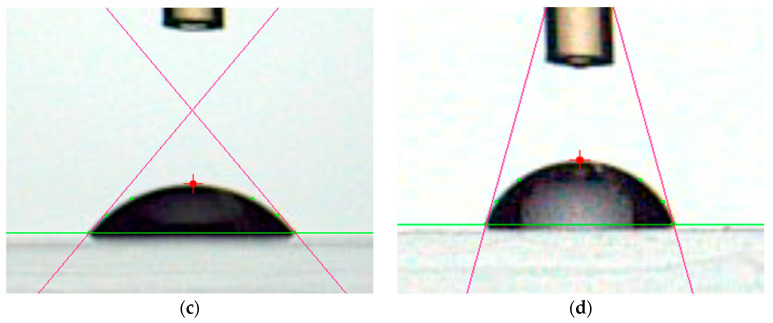
Photographs of contact angle measurement for PMMA films. (**a**) Untreated PMMA film, (**b**) annealed PMMA film, (**c**) plasma-treated (6 cycles) PMMA film, and (**d**) plasma-treated (6 cycles) and annealed PMMA film. The horizontal green line indicates the substrate surface. The perple line indicates the tangent line drawn to the contour (arc) of a water droplet in contact with the surface.

**Figure 6:**
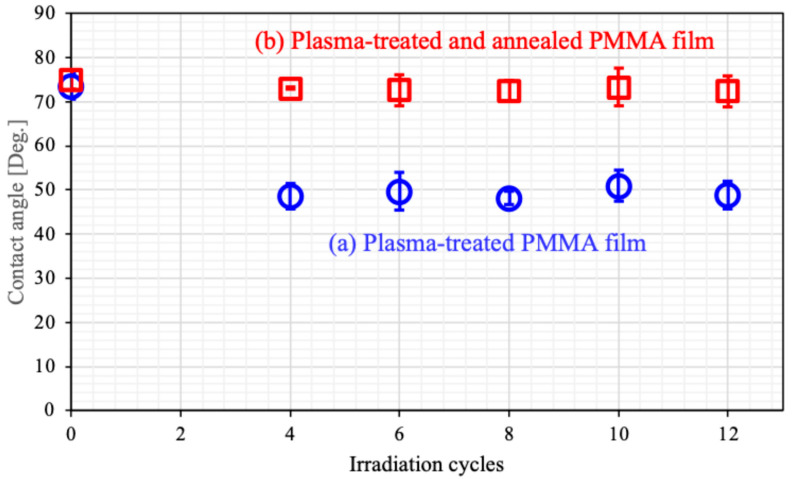
Contact angle dependence on plasma irradiation times for PMMA films. (**a**) Plasma-treated PMMA film (**○**, blue circle) and (**b**) plasma-treated and annealed PMMA film (**□**, red square).

Figure 5 presents photographs of contact angle measurements for PMMA films, while Figure 6 illustrates the dependence of the contact angle on plasma irradiation cycles. The surface treated only with plasma was hydrophilic. This hydrophilicity was almost independent from the surface roughness shown in Figure 3. Generally, hydrophilicity induced by microstructures is often explained using the Wenzel model [62]. The increased surface area by the formation of microstructures may be too small to affect wettability. In our previous work, we reported that the formation of microstructures results in super-nanohydrophilicity, similar to the surface of a snail’s shell [53,54,55,56,57]. In contrast, the wettability of the surface treated with both plasma and annealing recovered to that of the untreated surface. Gotoh et al. investigated the time course of wettability after plasma treatment and reported that hydrophobicity recovered [63]. One of our ideas is that annealing may promote this recovery. We also discuss the recovery of wettability with another idea later in this paper.

### 3.3. Antibacterial Test

**Figure 7:**
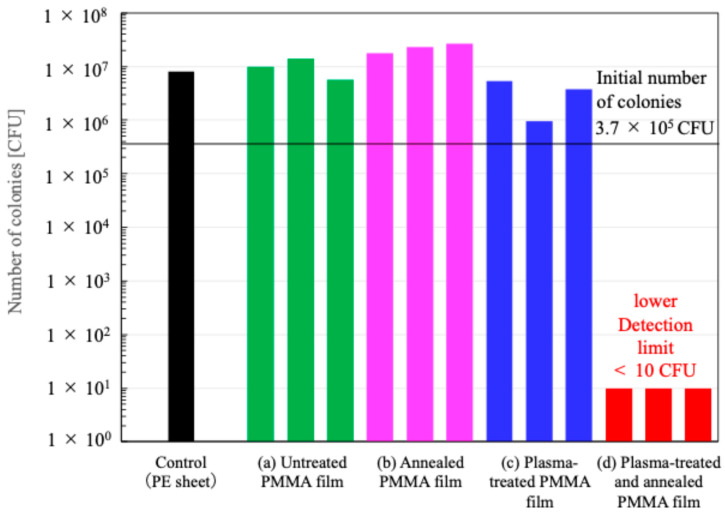
Number of colonies on the surface of PMMA films. The polyethylene sheet was used as a control (black bar). (**a**) Untreated PMMA film (green bars), (**b**) annealed PMMA film (magenta bars), (**c**) plasma-treated (6 cycles) PMMA film (blue bars), and (**d**) plasma-treated (6 cycles) and annealed PMMA film (red bars).

Figure 7 shows the number of colonies on the surface of PMMA films before and after the treatments. The number of colonies on the untreated substrate was comparable to that on the control sheet—polyethylene (PE) sheet. The number of colonies on the annealed film was similar to that on the untreated one. The average number of colonies on the plasma-treated film was 30% lower than that on the untreated one. Unfortunately, on these films, the number of colonies increased rather than the initial count. In contrast, the film annealed after plasma treatment exhibited a colony count at the detection limit (<10 CFU). This film achieved an antibacterial activity value greater than 5 with respect to the colonies of the untreated film. High antimicrobial performance was demonstrated. We discuss antimicrobial performance later in this paper.

## 4. Discussion

### 4.1. Thermal Shrinking

**Figure 8:**
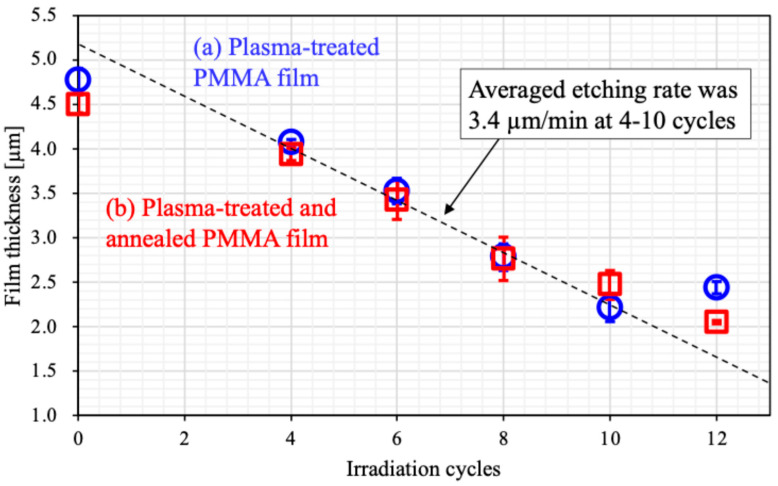
Relationship between the number of irradiation cycles and the film thickness. (**a**) Plasma-treated PMMA film (○, blue circle) and (**b**) plasma-treated and annealed PMMA film (□, red square).

Figure 8 presents the effect of PMMA film thickness on APLTP irradiation cycles for the plasma-treated PMMA film and plasma-treated and annealed PMMA film. The initial film thickness is represented by the value at zero irradiation cycles. Before annealing, the film thickness was 4.75 µm. After annealing, it decreased slightly to 4.50 µm. However, no horizontal shrinkage was observed. There was no peeling or warping of the film. The volume shrinkage was about 5% because the thickness shrinkage was about 0.25 µm. This reduction in thickness must be attributed to the shrinkage of the PMMA film caused by the heating during the annealing process. No further decrease in film thickness was observed after four or more irradiation cycles, both before and after annealing, suggesting that the surface side of the PMMA film may be more susceptible to shrinkage. The etching rate, about 3.4 µm/min, was determined for irradiation cycles ranging from 4 to 10, calculated based on the reduction in film thickness relative to the number of irradiation cycles. After 12 irradiation cycles, the observed film thickness deviated from the expected value based on the calculated etching rate. Between 10 and 12 cycles, a decrease in the etching rate was observed, perhaps indicating an increase in the density of the PMMA film at below 2 µm in thickness.

**Figure 9:**
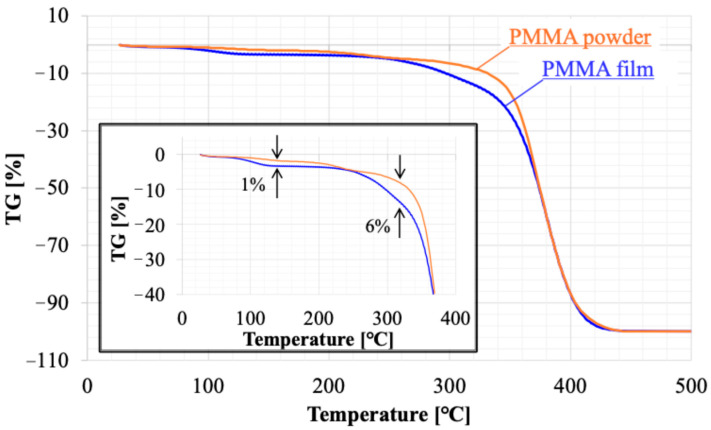
Results of thermogravimetry (TG) analysis; one is a raw powder sample of PMMA film (orange line) and the other is a sample recovered from PMMA film (blue line).

**Figure 10:**
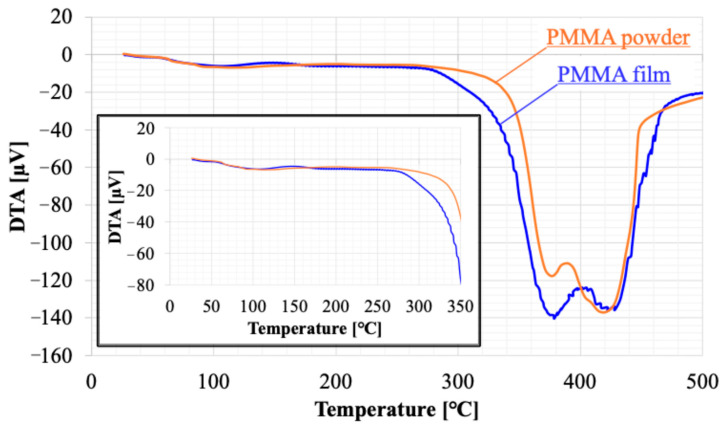
Results of differential thermal analysis (DTA); one is a raw powder sample of PMMA film (orange line) and the other is a sample recovered from PMMA film (blue line).

The etching rate remained slow for irradiation cycles between 0 and 4, which may be indicative of substances such as moisture and residue on the surface. Figure 9 and Figure 10 present the results of thermal analysis (TG-DTA) for a raw powder sample of PMMA film and a sample recovered from PMMA film. Weight loss was observed starting at approximately 80 °C. The DTA results suggested that this weight loss was due to an endothermic reaction. This phenomenon should be consistent across each sample because of the volatilization of adsorbed moisture. The weight loss stabilizes around 140 °C, with a total loss of 3%. The weight loss of the film sample was 1% more than that of the powder, suggesting that the film sample had more water adsorption sites. Above 360 °C, a sharp weight decrease occurred, which must be attributed to the thermal decomposition of PMMA. The temperature at which the weight loss reached −100% was the same for both, 440 °C. On the other hand, the weight loss between 260 and 360 °C indicates the presence of thermally decomposable substances within the film. DTA shows a large endothermic reaction that partially overlaps with the thermal decomposition of PMMA. Considering that the boiling point of ethyl lactate used as the solvent is 154 °C, the weight decrease observed at that temperature in the film sample must not be ascribed to the solvent remaining within the film; it must be due to the solvent material during the solvent casting process. It is a kind of solvent residue, and its weight is about 6% of the membrane weight.

### 4.2. Recovery of Wettability

The formation of microstructures by our proposed method should be distinguished from so-called surface roughening. Her et al. reported the formation of microstructures on PMMA surfaces using a focused Ga ion beam [64]. They explain that the formation of microstructures is caused by the penetration of Ga^+^ ions into the bulk of PMMA, and these interactions occur not only on the surface but also below the surface. Combining their explanation with our previous findings [53,54], we would like to consider the relationship between the formation of microstructures and wettability. Micropores, which are exit pathways for vaporized solvent molecules, may form both on the surface and inside the PMMA film. Dropped water percolates through the microstructures due to capillary action. The pores then provide air paths, allowing the water to fill not only the spaces between the spikes but also the internal pores of the film, leading to a decrease in the contact angle. We call this phenomenon super-nanohydrophilicity [53,55,56,57]. In contrast, the wettability of the surface treated with both plasma and annealing recovered to that of the untreated surface. This may be attributed to pore blocking, which is likely caused by the annealing. This is another idea regarding contact angle recovery. As mentioned above, the pores are thought to be formed by solvent evaporation and are distributed toward the surface. The inner side of the film must have fewer pores, which is attributed to the substrate side being warmer during solvent casting. The volume of film shrinkage by annealing should correspond to the volume of the blocked pores. As shown in Figure 8, the film shrinkage is about 5%, synonymous with volume shrinkage herein. The decrease in film thickness observed in the untreated PMMA film is likely due to the blocking of pores distributed toward the surface. As the film thickness decreases with increased plasma irradiation cycles, no further film shrinkage was observed. This may suggest that the pores are distributed toward the surface side of the PMMA film [53]. Here, we explain the behavior of dropped water when the pores are already blocked. Although water droplets attempt to percolate between the spikes and into the internal pores, unfortunately, the trapped air between the water and the spikes prevents further water penetration. This air pressure is well known as the Laplace pressure and is generally seen on hydrophobic surfaces [65]. These experimental facts can support the validity of our explanation.

### 4.3. Antimicrobial Performance

The bactericidal effects of both natural and synthetic nanopillars have been thoroughly reviewed by Tripathy et al. [9]. However, the complete elucidation of the fundamental mechanisms underlying microbial cell death on such surfaces has not yet been achieved. Several mechanisms have been proposed, but the contributions of factors such as wettability and surface roughness to the bactericidal activity of nanostructured surfaces remain subjects of ongoing debate [66,67,68,69,70,71]. Jindai et al. reported the wettability of nanopillars fabricated on Si substrates and the behavior of *E. coli* [72]. According to their study, the *E. coli* with motile flagella enhances adhesion to the hydrophobic nanopillar surface because of the hydrophobicity of its flagella. Bandara et al. reported that the movement of *E. coli* adhered to nanopillars damages the body of *E. coli* [10]. These previous studies may suggest the following antibacterial mechanism. *E. coli* with motile flagella is drawn to nanostructured surfaces due to the hydrophobicity of their flagella and the substrate. The *E. coli* adhered to the surfaces self-destructs due to the motility of their own flagella. Self-destructing *E. coli* becomes food for other *E. coli* and draws others to the surfaces. The *E. coli* that gathers there also self-destructs while eating and become food for the next *E. coli*. Linklater et al. reported that antibacterial performance differs depending on the type of polymer [16]. The antibacterial performance may be attributed to multiple factors, namely, the motility of *E. coli*, the hydrophobicity of the flagella, the hydrophobicity, the adhesiveness, and the base material. Our experimental results probably support the results of previous studies. The *E. coli* used in the antibacterial test is a wild-type strain, exhibiting flagella and chemical sensors. The nanospike structure, which was only plasma-treated, was hydrophilic and did not exhibit antibacterial activity. On the other hand, the nanospike structure, which was annealed after plasma treatment, was hydrophobic and exhibited excellent antibacterial performance, with an antibacterial activity value over 5 compared to the untreated substrate. Starting with this study, we would like to investigate more in detail the antibacterial performance against bacteria other than *E. coli* on various kinds of substrates with nanospikes.

## 5. Conclusions

In this study, we demonstrated the fabrication of dragonfly and cicada wing-inspired microstructures on polymethyl methacrylate (PMMA) films using atmospheric-pressure low-temperature plasma (APLTP) without relying on conventional lithographic processes. Plasma treatment successfully generated sharp-edged microstructures, while subsequent annealing modified the surface morphology and wettability by inducing densification and pore closure.

The plasma-treated surfaces exhibited increased hydrophilicity. After annealing, the wettability reverted to near-initial levels. Antimicrobial evaluation revealed that while only plasma treatment slightly reduced bacterial colonies, the film annealed after plasma treatment resulted in bacterial colony counts below the detection limit, indicating highly effective antimicrobial performance.

These findings highlight APLTP as a scalable and versatile approach for engineering functional polymer surfaces with antimicrobial properties, bypassing the material and geometric constraints of conventional methods. This work contributes to the advancement of biomimetic surface engineering and suggests a promising route for the practical development of antimicrobial coatings for large-area polymeric materials. Further research will focus on understanding the underlying bactericidal mechanisms and optimizing process parameters for specific application demands.

While the film with only plasma treatment reduced bacterial colonies by 30%, the film annealed after plasma treatment achieved an antibacterial activity value greater than 5, with bacterial counts below the detection limit (<10 CFU).

## Data Availability

Available upon request.
